# Comparing Distributions of Environmental Outcomes for Regulatory Environmental Justice Analysis

**DOI:** 10.3390/ijerph8051707

**Published:** 2011-05-24

**Authors:** Kelly Maguire, Glenn Sheriff

**Affiliations:** National Center for Environmental Economics, U.S. Environmental Protection Agency, 1200 Pennsylvania Ave., NW (MC 1809T), Washington, DC 20460, USA; E-Mail: maguire.kelly@epa.gov

**Keywords:** environmental justice, regulatory impact analysis, distributional analysis, equity, inequality index

## Abstract

Economists have long been interested in measuring distributional impacts of policy interventions. As environmental justice (EJ) emerged as an ethical issue in the 1970s, the academic literature has provided statistical analyses of the incidence and causes of various environmental outcomes as they relate to race, income, and other demographic variables. In the context of regulatory impacts, however, there is a lack of consensus regarding what information is relevant for EJ analysis, and how best to present it. This paper helps frame the discussion by suggesting a set of questions fundamental to regulatory EJ analysis, reviewing past approaches to quantifying distributional equity, and discussing the potential for adapting existing tools to the regulatory context.

## 1. Introduction

Economists have been interested in analyzing the distribution of environmental benefits for almost as long as they have been calculating the benefits themselves. While the tools for conducting benefits analysis are well developed, those for examining equity, or distributional effects, are less so.

Most OECD countries routinely perform a regulatory impact analysis of significant new environmental rules [1]. These analyses typically contain an estimate of monetized benefits and costs of options under consideration. They may also discuss how these benefits and costs are distributed across various subgroups, economic sectors, or regions. In the U.S., various Executive Orders (EO) require some distributional analysis (e.g., EO 13045 addresses children’s health, EO 13211 addresses energy issues). Relevant to this discussion, EO 12898, Federal Actions to Address Environmental Justice in Minority Population and Low-Income Populations, requires federal agencies to address “disproportionately high and adverse human health or environmental effects…on minority populations and low-income populations” [2]. To date, however, implementation of EO 12898 has been slow and inconsistent (see [3,4] for critiques of U.S. Environmental Protection Agency (EPA) implementation).

To be useful in the policy-making process, distributional analysis should facilitate the ranking of alternative outcomes. Such rankings are inherently normative, and thus should reflect the views of society as opposed the views of the technical staff preparing the analysis. There is a tradeoff. Purely descriptive analysis such as pollution exposure rates by subgroup may be difficult to digest and interpret in a consistent manner. However, methods for aggregating the data into easily presented rankings have the potential for implicitly reflecting staff value judgments. Ideally, the analysis would be prepared in a manner that is easy to understand yet flexible enough to allow normative judgments to be imposed explicitly.

In addition, for purposes of both decision-making and environmental justice there is a need for consistency and transparency. These concepts are related. Consistency implies that the decision-maker uses a similar framework to make decisions across rules. If a certain distribution of outcomes is preferred to another for one pollutant, then a similar ordering should be preserved for others. For the purposes of EJ, defined by the U.S. EPA to include “fair treatment and meaningful involvement,” transparency in decision-making is essential [5]. Interested parties should be able to identify the information and methodology used to make a decision is a way that is clear and accessible. In identifying methods for use in EJ analysis for regulatory policy we are cognizant of the need for both consistency and transparency.

Here, we present various methods used in the (mostly) economics literature to quantify the distribution of environmental impacts, and evaluate their usefulness through the prism of how the results can be used to guide the environmental regulatory process. The few examples discussed here are not intended to be comprehensive (for recent reviews of the EJ literature see [6–8]). We begin Section 2 with a discussion of three fundamental questions that a distributional analysis of environmental policy options could address. In Section 3 we describe efforts in the literature to describe environmental or health outcomes for different subgroups. Since the objective of most of these studies is to describe existing distributions, we discuss how they may be adapted to the purpose of evaluating prospective policy options. In Section 4 we describe methods (Lorenz curves, concentration curves, and inequality indices) to aggregate this information in a way that allows one to rank policies in a transparent and consistent manner. In Section 5 we offer concluding thoughts and some potential steps forward.

## 2. Three Fundamental Questions for Regulatory EJ Analysis

Environmental justice is a concern that certain subgroups, typically defined by race or income, have historically borne a disproportionate share of environmental burdens. In the context of new regulations it is important to outline a consistent set of questions a distributional analysis of environmental policy could address.

With regulatory impact analysis the primary concern is distributional effects associated with options under consideration, as opposed to the causes of inequities typically investigated by the academic literature. The goal is to provide the decision-maker and public with information regarding the degree to which regulatory options under consideration remove or worsen previous disparities in environmental outcomes for vulnerable communities, or create new disparities where none existed. As such, it is important to analyze changes in distributions of environmental outcomes between baseline and various policy options, rather than just the distribution of changes (since an unequal distribution of environmental improvements may actually help alleviate existing disparities).

Before turning to the questions to guide the analysis, it is important to identify the outcome to be measured. Options include pollution (e.g., parts per million of ozone), health effects (e.g., number of cases of asthma), and monetized benefits (e.g., willingness to pay for reductions in asthma cases). Here, we adopt the position dominant in the environmental justice community (if not the economic literature) that the distribution of physical outcomes (e.g., pollution or health effects), rather than their monetized value is most appropriate for regulatory analysis.

Methods for attributing monetary value to environmental outcomes (such as health impacts) typically employ measures of individuals’ willingness to pay for a small improvement in environmental quality. These monetary values can be used to analyze the distribution of changes in environmental outcomes, but are not useful for comparing distributions of outcomes before and after a policy intervention. Such a comparison would require individuals’ total money-metric utility (*i.e.*, not just the value of the change in utility), which current techniques generally do not calculate (for an overview of methods for monetizing environmental outcomes, see [9]).

We also focus exclusively on the distribution of environmental outcomes, not the distribution of economic costs (e.g., higher prices or reduced employment) associated with a particular regulatory option. For a recent survey of the economic literature analyzing the incidence of the costs of environmental regulation (primarily by income group), see [7]. Whether to use pollution or health effects depends on data availability. Since they most directly affect human well-being, health effects are the most relevant outcome. When this information is unavailable, pollution exposure levels may be a useful proxy, followed by ambient pollution concentrations, plant emissions, and proximity to a source [10,11].

It is useful for the analysis to begin with an understanding of the baseline distribution of the environmental outcome of concern:

### 

#### (1). What is the baseline distribution of the environmental outcome?

Establishing a proper baseline distribution is crucial for two reasons. First, identification of a pre-existing disparity presents an opportunity to tailor policy options to address the disproportionate impact directly. Second, the baseline establishes a marker for determining distributional impacts of the policy itself. Once the baseline has been established, it is useful for the analysis to predict the ex-post distributional effects of the regulatory options under consideration.

#### (2). What is the distribution of the environmental outcome for each regulatory option?

While the options under consideration may be implemented uniformly (e.g., the same standard would apply to all individuals, geographic locations, or types of facilities), the distribution of the pollutant in the predicted post-regulatory scenarios may differ for several reasons. First, the type of regulation may affect the post-regulatory distribution. For example, a uniform rate-based standard (per unit of output) means that facilities with higher output will generally have higher post-regulatory emissions. Second, to the extent that different types of individuals (e.g., low-income) have different sensitivities to a given pollutant or different exposure pathways, some individuals will experience a different post-regulatory scenario than others. Answering this question for prospective options requires the capacity to model alternative outcomes. Finally, it is important to assess the degree to which various policy options create or remove disproportionate impacts.

#### (3). How do the policy options being considered improve or worsen the distribution of the environmental outcome with respect to vulnerable subgroups?

Answering this question requires a methodology for comparing the answers to the first two questions in order to determine whether a regulation represents an improvement to the status quo and other considered options, and ideally an indication as to how much.

Responses to these three questions can be presented in conjunction with net benefits arising from the policy options. This combination of information would enable policy makers to understand the possible tradeoffs between environmental justice and overall economic efficiency implicit in the decision-making process. It is important to note that there may be limited opportunities within the policy design itself to address any post-regulatory distributional effects. Regardless, clear documentation and acknowledgment of those effects is informative to the decision-maker and the public, and may help guide future policy.

These three questions provide a basic framework to inform the distributional analysis for environmental regulatory policy. This framework also enables analysts to identify if and how existing disparities may be addressed through the regulatory context, recognizing that legal, political, and enforceability constraints may prevent any action in this regard.

Note that such an analysis may not always be feasible. Data constraints may prevent the identification of existing or post-regulatory disparities. The geographic distribution of the pollutant may be unknown, for example. While advances in air monitoring and modeling allow for more detailed assessments of how pollutants are dispersed, such analytical efforts require significant time and resource allocations. Some water pollutants are even more problematic as little is known about the fate of a pollutant after discharge.

Related to the issue of data constraints is the fact that both pollution dispersion models and demographic information are imperfect. Regarding pollution there is uncertainty in the models or monitoring or sampling used to generate baseline and control scenarios. The quality of the data is also likely to vary across pollutants. With respect to demographic information, data such as income levels are typically publicly available only at an aggregated level. The U.S. Census, for example, reports median income at the block group level. As it is beyond the scope of this article to develop tools for incorporating uncertainty, decision-makers’ risk preferences, and other practical implementation issues into the distributional analysis, we leave these topics for future research. Other authors have examined related issues, however. Hubbell *et al*. [12], for example, discuss the role of error in air pollution dispersion models. For a discussion of methods for incorporating sampling error into inequality index analysis, see [13–15]. For a methodology to address the bias introduced by assigning median income to all residents of a Census block group, see [16].

Moreover, answering these three questions is by no means sufficient for addressing all EJ issues. For example, analysis that focuses on a single pollutant typically does not account for the contribution of cumulative effects from other pollutants or multiple exposures from sources outside the scope of the proposed rule. Disproportionately affected communities may suffer from multiple stressors that have accumulated over decades. One specific pollutant may show little impact or may even be distributed fairly evenly. In an area with multiple waste sites or polluting facilities, however, the marginal effect of a particular pollutant may be greater than in a community without such stressors.

Related to this point, analysis focusing on pollution concentrations or exposure levels, rather than health outcomes may also fail to account for baseline differences in health risks across racial and ethnic groups and income categories. Such differences may exist due to genetic, cultural, or other un-accounted factors. There is increasing evidence that the same exposure affects people differently, and those effects can vary along racial and ethnic lines and socioeconomic status. In addition, individuals with low incomes have less access to averting behaviors and resources, like medical care, alternative water sources, or housing options that allow them to avoid exposures. Thus, assuming that exposure affects everyone in the same manner may be misleading.

With these caveats in mind, we now discuss ways to present information in a way that is helpful for addressing these three questions.

## 3. Describing Distributions

A tradeoff exists between providing information in a way that is useful to policy makers and imposing ethical assumptions on the part of the analyst. This section describes quantitative methods that have been used to describe the distributional effects of various environmental outcomes with a minimum of ethical input.

Distributional effects are quantified in a variety of ways in the academic literature. While a consensus has not been reached on how best to analyze, quantify, and present the results of an environmental justice analysis, a suite of methods has emerged over the last few decades that can be categorized as visual displays, summary statistics, and regression results. The variation in methods both within and across these categorizes can be attributed to author preference or expertise, as well as the research question at hand. In this section we survey key methods for quantifying distributional effects and evaluate their effectiveness in addressing the policy questions outlined above.

### 3.1. Visual Displays

The use of charts, graphs, and maps can be useful to provide an overview of the data and results used in analysis. Beginning with the earliest study in our review, Dorfman [17] examines the distribution of benefits and costs of environmental programs. Results are shown graphically as a percent of household income. Shadbegian *et al.* [18] reported one of the few distributional analyses of a specific rule. They show the distribution of monetized benefits and costs from the SO_2_ trading program across U.S. regions. Results are presented using tables and maps.

The graphical displays, as well as those that use maps to present information (e.g., [18–21]) are a useful complement to other quantifiable information. Geographic Information System generated maps are useful for suggesting trends, showing the general location of where pollution is greatest or disparities are most pronounced. However, in terms of analyzing the baseline or ex-post distribution of pollution, such displays are suggestive at best, and lack the level of detail required in a decision-making context. In particular, they can be effective at conveying differences between baselines and policy options if the differences are stark. For more subtle changes, however, they are less useful.

### 3.2. Summary Statistics

Summary statistics are a key component of any empirical analysis, providing the reader with an important overview of the data used in the study. These statistics typically include information on the number of observations associated with a particular variable, some measure of central tendency, such as the mean or median, and a measure of dispersion, such as the standard deviation. Although they are quite simple, these statistics can provide useful insights into the patterns of disparities regarding environmental outcomes. In addition, summary statistics can be applied consistently across regulatory scenarios and are typically transparent to the reader. Information on the quantity of a particular pollutant across income quintiles or racial groups, for example, gives insight into whether or not the pollutant is evenly distributed, and this may be accompanied by some measure of statistical significance. With respect to the questions outlined above, these statistics are useful for establishing baseline incidence of environmental burdens, and can be used to measure both post-regulatory incidence and changes in incidence.

Asch and Seneca [22] and Harrison and Rubenfield [23] are two early studies of the distribution of pollution in the U.S. Both studies examine the distribution of air pollution across various demographic variables, including income and race. Relevant for the policy questions we pose, the authors analyze both the baseline and the changes in air pollution due to current regulations. Asch and Seneca [22] find that the baseline distribution of particulate matter was regressive. Using the correlation between seven categories of income and particulates in 284 U.S. cities they find that z-statistics show a positive correlation for the lower income groups and that regulations helped ameliorate these effects.

Harrison and Rubenfield [23] show baseline and control scenario exposure to NOx concentrations for seven income groups in Boston. They show the concentration levels across the income groups for the baseline and control scenarios and make some qualitative statements about the results (e.g., the distribution of baseline concentrations is fairly even across income groups, but the poor receive more benefits from reductions).

More recently, Brajer and Hall [24] examine changes in ozone and particulate matter with respect to various demographic variables for the Los Angeles basin for 1990–1999. The data are presented as “population weighted pollution levels” by county, race and income. A Spearman rank correlation analysis shows correlation between pollution and socio-economic variables. They find that pollution has fallen over the decade in the region, but the air quality gains are not evenly distributed.

While this brief review is not comprehensive, it provides a sense of the type of information summary statistics convey in the literature. The methods are straightforward and easily understood, and are useful for answering the first two questions in Section 2. They provide useful baseline information regarding outcomes across subgroups, as well as the correlation between group characteristics and environmental outcomes. When combined with models that predict pollutant responses, they could provide similar information for alternative regulatory options.

Summary statistics are unlikely to contain sufficient information regarding the third question, however. They are not useful for evaluating the relative merits of regulatory options (including the status quo) since they do not reflect distributions within subgroups. Such information can be important since the impact of a pollutant may be more of a concern if it is concentrated in a hotspot among a relatively small group of individuals than if it is evenly spread across the sub-population. In such situations, focusing on averages or correlations can be misleading since a low average exposure may mask very high exposure for a subset of individuals within a group. There may be an undetected EJ problem if such hotspots occur primarily in vulnerable subgroups.

In addition, these statistics do not provide a clear, systematic ranking of alternatives. Different policy options may involve tradeoffs between total improvements across all groups and reducing the disparities among groups. Simple averages or correlations provide no guidance regarding a transparent way to resolve these conflicts within one regulatory analysis, much less consistently across rules.

### 3.3. Regression Analysis

Regression analysis is a cornerstone of empirical economic analysis. It allows researchers to use data to provide internally consistent, unbiased hypothesis testing. In terms of environmental justice, regression analysis is frequently used to identify the existence and causes of various environmental outcomes across subgroups. By controlling for confounding factors, researchers can identify impacts of key independent variables on measures of interest. There are numerous ways to conduct regression analysis in the context of EJ; here we highlight a few.

A common framework is to use a probability-based model to account for the fact that not all locations experience a particular outcome (e.g., toxic releases or facility siting), and there may be systematic differences between areas with and without the release. Baden *et al.* [25] conducted an analysis of Superfund sites using a logit model and control for location characteristics, such as population density, population size, and state fixed effects. Results show a significant and positive relationship between the percent Black and Hispanic and the probability of having a Superfund site, and that the higher the income the less likely the area has a site.

Downey *et al.* [26] examine toxicity-weighted U.S. air pollution Risk-Screen Environmental Indicators data and their distribution across race and ethnicities. The authors assign each of six race and ethnic groups within metropolitan areas a score based on exposure to air pollution. They use a logit model to examine how income affects the probability of receiving a high score, controlling for community characteristics, such as density, employment, region, *etc*. They find a strong link between income and disparities in releases across 329 metropolitan areas, but the link with race is less significant.

Wolverton [27] uses a conditional logit model to examine plant siting decisions by using of community characteristics at time of siting, rather than after construction. This distinction is important since facility siting can cause housing prices or wages to change in affected areas, which in turn can lead to migration that alters a location’s demographic characteristics. Controlling for several variables including property values, wage rates, education, employment, *etc*., she finds that income, but not race, affects location decisions.

Arora and Cason [28] use a Tobit model to examine the effect of neighborhood characteristics on Toxics Release Inventory emissions by ZIP code for 1990. They first estimate the probability that a geographic area has a facility with releases, and estimate the size of the release in a second stage. The authors find that there is a significant coefficient on race variables in the Southeast. The coefficients suggest that areas with more non-white residents are more likely to have higher emissions. Income follows an inverted U-pattern; emissions initially increase with income until reaching a point after which emissions fall as income rises.

Fowlie *et al.* [21] use a difference-in-difference approach to examine the relationship between emissions of facilities participating in the California Regional Clean Air Incentives Market and demographic variables. Their model allows them to examine emissions before and after implementation of the emission trading, controlling for county attainment status, community, and demographic variables. They compare effects of the trading policy with the counterfactual of traditional command and control regulation. They find that neighborhood demographic characteristics are not a statistically significant predictor of changes in emission levels.

In general, regression analysis is useful for teasing out causal factors behind relationships between socio-economic variables and environmental outcomes. However, for purposes of an EJ regulatory analysis most (with the exception of [21]) do little to inform the question of baseline and post-regulatory scenarios. Conducting careful regression analysis is time and data intensive. Consequently, it is likely to be beyond the resources available for regulatory impact analysis. Moreover, while studies such as [21] are able to indicate effectiveness of race or income as a predictor of emissions for different policy alternatives, they are not designed to rank these alternatives.

## 4. Ranking Distributions

While the methods described in the previous section are useful for addressing many important questions, they do not rank outcomes in a way that answers our third question in a transparent manner. Fortunately, a set of tools for ranking distributions is relatively well developed in the context of income and health outcomes. The literature on applying these methods to rank environmental policy outcomes by their distributional impacts is still in its infancy, however.

In this section, we outline how this literature has been adapted to address environmental justice questions, identifying some shortcomings and suggesting some steps forward. We begin with a set of visual ranking tools, Lorenz and concentration curves, which allow one to determine easily if one distribution of outcomes is more “equitable” than another. These tools are only applicable, however, for a small set of possible distributional comparisons.

We then discuss several inequality indices, the Gini coefficient, the concentration index, the Atkinson index and the Kolm-Pollak index. Unlike the visual ranking tools, these indices permit the analyst to rank any set of distributions. This universal applicability comes at the expense of imposing additional normative assumptions, however. This tradeoff can be most easily seen with the Gini coefficient and concentration index. Although these two indices can be derived respectively from the Lorenz and concentration curves, they do not provide identical information as the curves. The indices can rank distributions that the curves cannot, but they require the analyst to impose stronger normative restrictions.

### 4.1. Visual Ranking Tools

We begin with two visual ranking tools, the Lorenz curve and the concentration curve. These tools have the advantage of imposing relatively few ethical standards on an ordering; however, they are unable to provide a complete ranking of distributions. In addition, they do not provide much useful information regarding distribution of environmental outcomes across subgroups, limiting their applicability to EJ analysis.

**Figure 1.**
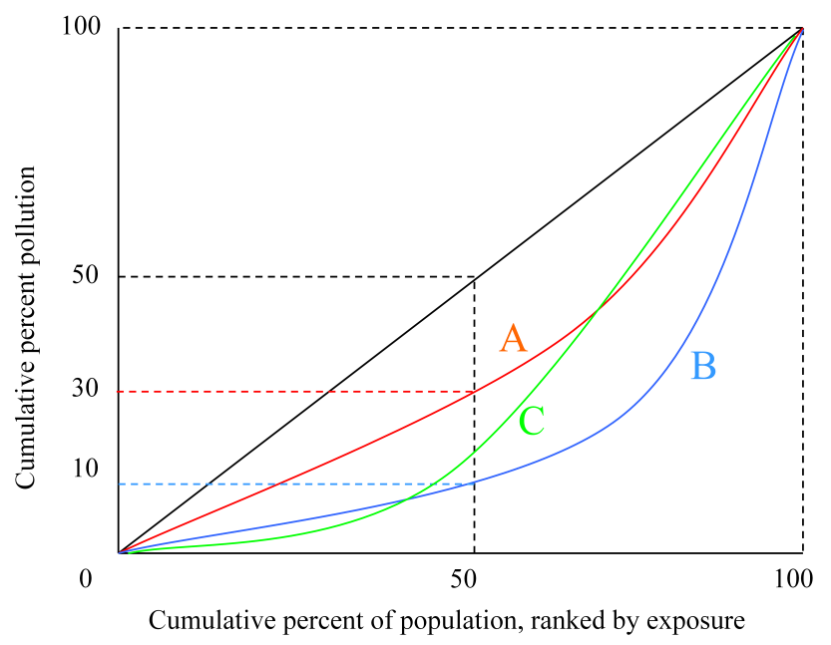
Lorenz curves.

**Lorenz Curves.** If one accepts the ethical premise that it is always desirable to transfer a unit of pollution away from a highly exposed individual to a lesser exposed one, then Lorenz curves provide a means of ranking policy outcomes. Some hypothetical Lorenz curves for distribution of a pollutant are depicted in Figure 1. The horizontal axis of the graph indicates percentiles of the population ranked by pollution exposure: 10 corresponds to the ten percent of the population least exposed to the pollutant, 50 corresponds to the half of the population least exposed to pollution, *etc*. The vertical axis represents the percent of pollution exposed by percentile. The black diagonal line depicts a perfectly equal distribution of exposure: the lowest 10 percent of the population experience 10 percent of the exposure the lowest 50 percent of the population experience half the exposure, *etc*.

Curves A, B, and C represent three hypothetical Lorenz curves in which pollution is not distributed equally. In curve A, for example, the least exposed half of the population is exposed to 30 percent of the pollution, while in curve B the least exposed half experiences only 10 percent of the pollution. Lorenz curves have the useful feature that the farther away the curve is from the diagonal, the less equal is the distribution. This property can form the basis of a ranking system. Suppose A and B represent the predicted distributions of two regulatory options. For now, let us suppose that the two policies result in the same amount of pollution per capita. Option A results in a more equitable distribution than Option B. The only value judgment that needs to be imposed to make a preference ranking is that one care at all about distributional equity. It does not matter how much one cares about exposure at the top or bottom of the distribution. As long as one prefers a more equal distribution to a less equal one, a curve that is closer to the diagonal (such as A) is preferable to a curve that is farther (such as B).

Although Lorenz curve analysis imposes minimal value judgments on the part of the analyst, it has several drawbacks that limit its practical usefulness. First, it is only a partial ordering, meaning that it can only draw meaningful comparisons for options whose Lorenz curves do not cross. A policy generating curve C, for example, cannot be compared with curves A and B since it is closer to the diagonal for some range of the population, but farther for others. This property is particularly problematic if one is interested in several options since the more curves being analyzed the more likely that some will cross.

Second, Lorenz curve analysis is ordinal; one can say that A is preferred to B, but not by how much. This ordinal property is related to a third issue. Lorenz curve analysis ignores differences in average exposure levels. For example, if we abandon the assumption that each distribution has the same average pollution level, the exposure levels of the most highly exposed individual in distribution B may be lower than the least exposed in distribution A. It may be undesirable to conclude that A is preferred to B simply because the exposure is more equitably distributed. Lorenz curves do not provide any means of evaluating a tradeoff between lower average exposure levels and a less equitable distribution. (The generalized Lorenz curve developed by Shorrocks [29], however, does allow a partial ordering of distributions with different means.)

Finally, for purposes of environmental justice analysis, Lorenz curves have the shortcoming that they are not easily disaggregated by population subgroups. It is straightforward to use Lorenz curves to compare distributions of pollutants within a sub-group (e.g., define the population and exposure percentiles in terms of individuals below a poverty threshold). It is not so easy to use Lorenz curves to evaluate distributions across subgroups (e.g., to make statements to the effect that a regulation causes pollution to be more equitably distributed across racial groups). Although Lorenz curves can be decomposed by subgroup [30], this decomposition does not allow one to rank distributions as in the aggregate Lorenz curve analysis.

**Figure 2.**
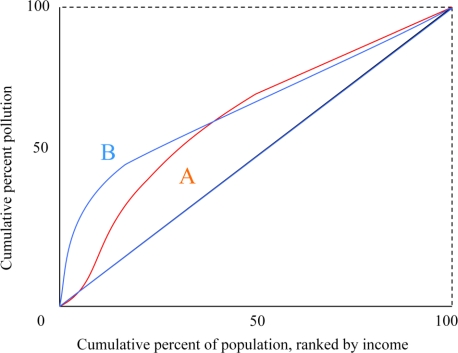
Concentration curves.

**Concentration Curves.** Like the Lorenz curve, the vertical axis of the concentration curve displays the share of an outcome variable experienced by a population. The horizontal axis displays the cumulative percent of the population ranked by socio-economic status (typically income). A Lorenz curve, in contrast, would display the population ranked by exposure. The height of the concentration curve indicates the share of the outcome experienced by a given cumulative proportion of the population. Figure 2 displays hypothetical concentration curves. A perfectly equal distribution of outcomes corresponds to a concentration curve along the 45° line. Kakwani [31] first developed this analysis to study income tax progressivity. Wagstaff *et al.* [32] proposed its use in measuring the equity of health outcomes.

Unlike Lorenz curves, concentration curves can cross the 45° line, and even lie completely above it if lower income is correlated with higher outcomes. Concentration curves can rank distributions in a manner similar to Lorenz curves; for a good outcome, a higher curve is socially more desirable. Concentration curve rankings implicitly employ social preferences such that it is always desirable to transfer a good environmental outcome away from a relatively rich individual towards a poorer one, even if the poorer individual is slightly poorer and significantly healthier [33]. Note that this normative judgment may be more controversial than the corresponding assumption used for Lorenz curve analysis (that it is socially desirable to shift good health outcomes to the relatively ill).

Concentration curve analysis suffers from the same shortcomings as Lorenz curve analysis. It is unable to rank distributions whose curves cross, thus providing only a partial ordering. It is ordinal, and ignores differences in average exposure levels. It is also unable to evaluate changes in distributions between subgroups (other than those based on income).

In general, both visual ranking tools have some advantages over the visual displays discussed in the previous section. In some cases, both Lorenz and concentration curves allow comparisons across policy alternatives. In addition, concentration curves provide information regarding equity of an environmental outcome with respect to one demographic variable of interest, income. However, both curves share the main shortcomings of the other visual displays; they are only effective at comparing distributions if there are sufficiently stark differences. If the curves for different policy options cross, this analysis provides no effective ranking methodology.

### 4.2. Inequality Indices

An inequality index is a mathematical tool for converting a distribution into a single number. That number can then be used to generate an ordering for any set of outcomes, thus addressing the partial ordering issue inherent in the Lorenz and concentration curve analyses. For example, a distribution with a higher inequality index number is less equal, and hence less preferred than one with a lower number. Moreover, some inequality indices can be decomposed in a manner that allows one to evaluate inequality both within and between subgroups of interest. An index value can also have cardinal (rather than just ordinal) significance, *i.e*., the magnitudes, not just the rankings, contain useful information. However, these useful features come at the cost of imposing subjective value judgments. In addition, their usefulness for evaluating distributions of bads can be problematic.

Here, we focus on four families of inequality indices: the Gini coefficient, the concentration index, the Atkinson index, and the Kolm-Pollak index. For a discussion of other index numbers in the context of income distribution, see [34]; in the context of environmental outcomes, see [10]. These indices can be divided into the categories of relative (Gini coefficient, concentration index, and Atkinson index) and absolute (Kolm-Pollak index) indices. Relative indices are unaffected by proportionate changes in the outcome variable. They are therefore convenient for analysis of variables using different units of measurement (e.g., currencies for income analysis). In contrast, absolute indices are unaffected by a uniform shift in the outcome variable (*i.e.*, the addition of a constant to every individual’s outcome). These properties are mutually exclusive, and there is no unambiguous reason to choose one category of index over another. As argued by [35], however, relative indexes can be misleading. Suppose the income of both members of a population of two individuals doubles. If prices do not change the difference in purchasing power between the two would also double, suggesting that the new distribution is less equal. An absolute inequality index would increase to reflect this change, while relative index would not.

Blackorby and Donaldson [36,37] show that relative and absolute indices that depend only on one variable have an associated ordinal social evaluation function (the proofs do not apply to the concentration index since it depends on two variables, environmental outcome and income). The equally distributed equivalent (EDE) value of a distribution is the amount of the outcome variable that, if given equally to every individual in the population, would leave society just as well off as the actual, unequal distribution. The EDE thus embodies a set of social preferences and is a measure of social welfare that enables rankings of distributions with different means. The Gini coefficient, Atkinson index, and Kolm-Pollak index can all be expressed as functions of their associated EDEs.

Choosing a specific type of index with which to rank policies is thus equivalent to choosing a particular social evaluation function on which to base the policy decision. Since the values of the associated social evaluation function do depend on the average value of the outcome variable (not just the distribution), they provide an additional tool with which the analyst can compare policy outcomes that differ in both mean and distribution in a logically consistent manner.

Although the social evaluation functions are ordinal, the associated inequality indices are cardinal. A relative index answers the question, “What percent of the average amount of the good would society be willing to sacrifice if the remainder were allocated evenly across the population?” An absolute index answers the question, “What is the amount of the good per capita society would be willing to sacrifice if the remainder were allocated evenly across the population?” Thus, magnitudes, not just ranking of the indexes are significant.

**Gini Coefficient.** The Gini coefficient is the most widely used inequality index. Its popularity is likely due more to the fact that it is easily understood as an increasing function of the area between a Lorenz curve and the diagonal line representing perfect equality than to desirable theoretical properties. The Gini coefficient has the undesirable feature that the effect of a transfer on the index number depends on the individuals’ ranks, not the difference in outcomes. In contrast to the widely accepted principle that an inequality index should place greater weight on transfers among the relatively worse off, for a typical bell-shaped distribution a transfer between individuals in the middle of the distribution will have a higher effect on the Gini coefficient than a transfer between two similarly distanced individuals at either tail [38]. There are ways of modifying the Gini coefficient to introduce flexibility in the weights placed on different segments of the population [39,40]. These techniques are rarely used in practice, however.

The Gini coefficient also has the undesirable property that the effect of a transfer on the index depends on the endowment of a third individual; if that individual is ranked between the first two, the transfer will have a greater impact than if not (since there will be a greater rank difference between the first two individuals in the former case). Finally, and particularly troublesome for EJ analysis, the Gini coefficient cannot generally be used to decompose aggregate inequality into within and between group components in an internally consistent manner [34]. Specifically, constructing an EDE for each subpopulation and then using these to construct an aggregate EDE for the entire population does not yield the same result as calculating the aggregate EDE directly.

Although it is a simple matter to compute a Gini coefficient if the outcome of concern is a bad (rather than a good), the resulting measure does not have a sensible associated social evaluation function (since it would be increasing in the bad). It is an ordinal ranking of dispersion, but loses the cardinal interpretation of a relative inequality measure since the EDE is smaller than the mean (for a bad it should be larger). Thus, it does not indicate the percent increase in average pollution that could be tolerated in exchange for a perfectly equal distribution. Consequently, the Gini coefficient can provide useful comparisons for distributions with the same mean level of a bad, but cannot be used in conjunction with a social evaluation function to rank distributions with different means. Moreover, using the Gini coefficient in this way can be misleading since it can generate different policy rankings if one uses a bad as the outcome variable versus its complementary good. Calculating the Gini coefficient for ambient concentrations of parts per billion of an air pollutant, for example, yields a different ranking of policy outcomes than using the same data to calculate a Gini coefficient for parts per billion of “clean” air.

There are several examples of applications using the Gini coefficient to analyze distributions of health and environmental outcomes. Among the first were [41], who used a Gini coefficient to track evolution in age at death (a good) over time in Great Britain. Heil and Wodon [42] use a Gini coefficient to examine the distribution of predicted CO_2_ emissions across countries grouped by income. Millimet and Slottje [43] use the Gini coefficient to compare distributions of pollution across states grouped by income class. Since the Gini coefficient does not satisfy consistency in aggregation both of these studies required a group overlap term in addition to between and within group terms. Millimet and Slottje [44] use the Gini coefficient to evaluate the effect of regulatory compliance costs on the distribution of toxics reported in the U.S. Toxic Release Inventory across U.S. states and counties. They combine regression results with Spearman correlations between demographic characteristics and emissions to argue that policies that increase inequality as measured by the Gini coefficient increase racial disparities. In these studies, the Gini coefficient has been used primarily as an ordinal measure of dispersion, without attendant welfare implications.

**Concentration Index.** The concentration index is similar to the Gini coefficient, being an increasing function of the difference between the 45° line and the concentration (rather than Lorenz) curve. For details on the practical use of the concentration index, see [15]. Its value ranges from −1 (the entire outcome is borne by the poorest individual) to 1 (the entire outcome is borne by the wealthiest individual). Since the concentration curve can cross the 45° line, zero either indicates perfect equality or that the area above the curve is exactly equal to the area below it. As with the Gini coefficient, the effect of allocating a unit of the outcome variable to an individual is weighted by the individual’s rank. With the concentration index, the relevant rank is income, rather than the outcome variable.

The concentration index can provide a complete ordering in the sense that lower values are always more “pro-poor” (for distribution of a good) than higher values. The cardinal relationship between magnitudes of concentration index numbers lacks the clear intuition of the other three indices considered here, however. This is not to say that there is no intuitive interpretation. Koolman and van Doorslaer [45] provide a link between the index value and the proportionate amount of the outcome variable that would need to be redistributed from the richest to the poorest half of the population in order to attain an index value of zero (not necessarily equality).

Like the Gini coefficient, the concentration index value depends on individuals’ ranks, not absolute differences. It also shares the trait that ordering based on the concentration index can be sensitive to whether the outcome variable is expressed as a good or its “bad” complement [46]. It inherits from the concentration curves the questionable normative assumption that transfers of a good environmental outcome from rich to poor is always desirable [47].

**Atkinson Index.** The Atkinson index satisfies several desirable theoretical properties lacking in other relative indices [35,36,38]. Among these are that it is a function of individual allocations rather than rank, and it can be disaggregated into subgroups in a consistent manner (see also [48]).

In its formula, the Atkinson index explicitly incorporates ethical considerations with an inequality aversion parameter that ranges from zero to infinity. This parameter introduces some flexibility, allowing the analyst to specify the amount society is willing to trade a reduction in the outcome variable for one individual for an increase for another. A value of zero implies that society is indifferent between transfers between any two individuals. The higher the parameter’s value, the more weight society places on transfers to individuals with lower outcomes. Since the choice of a parameter value is entirely normative, it is common to calculate Atkinson indexes for several values to determine how sensitive rankings are to the choice.

Although the Atkinson index has many desirable properties when used to analyze distributions of goods, it is not so convenient for analyzing bad outcomes. As with the Gini coefficient, inputting a bad into the Atkinson formula removes any cardinal welfare significance since the associated social evaluation function would be increasing in the bad. It also causes the index to place more weight upon the most well-off individuals (those with low outcomes), rather than the worst off. The Atkinson index is generally not defined for negative numbers, thus precluding a simple redefinition of bads in that way. Even for examples in which negative values are defined, the Atkinson Index generates the perverse result that a progressive redistribution reduces social welfare [49].

Transforming a bad into a good by replacing it with its complement (e.g., parts per billion of a pollutant to parts per billion of “clean” air, or the probability of not dying from cancer) may have the undesirable result of rendering an index value so small as to be within rounding error. To put this in perspective, consider the relative income distribution of a society of billionaires who differed in wealth by only a few dollars. It would be almost perfectly equal, with the value of the corresponding Atkinson index being extremely close to zero. Note that this does not mean that the distributional effects are insignificant. If the good were clean air or probability of not dying from cancer the percent reduction society would be willing to give up for an equal distribution might be quite small, but the value of that reduction might be significant. Nonetheless, presenting the results in a manner such that a regulation changes the Atkinson Index by a miniscule amount may not be easy to interpret.

Although the Atkinson index is commonly used in income distribution analysis, it has rarely been used to measure environmental or health outcomes. Waters [50] used an Atkinson index to analyze distribution of access to health care (a good) in Ecuador. Levy *et al.* [20] used the Atkinson index to evaluate the distribution of mortality risk resulting from alternative power plant air pollution control strategies in the United States. Levy *et al.* [51] used the Atkinson index to analyze reduction in mortality risk from particulate matter reductions from regulating transportation. Each of these studies used the Atkinson index as a measure of dispersion without welfare significance.

**Kolm-Pollak Index.** The Kolm-Pollak index shares the desirable theoretical properties of the Atkinson index [35,37,48]. It also uses an inequality aversion parameter to specify the relative importance of allocations to different segments of the population. Higher values correspond to greater weight being placed on the worse off and zero indicates complete indifference to the allocation.

In contrast with the other indices examined here, the Kolm-Pollak index readily accommodates bad outcomes. It is inappropriate to input bad values directly into the index. However, one can simply multiply them by minus one and add them to some arbitrary benchmark. This operation preserves the appropriate social evaluation function ranking and is equivalent to measuring the distribution of a complementary “good.” The property of an absolute index that adding the same amount to everyone in the population does not change its value helps in this regard; the value of the index is independent of the benchmark level. To date, the Kolm-Pollak index has not been used in the analysis of environment or health outcomes, and there are few examples of its application in income analysis (an exception is [52]).

In general, the Atkinson and Kolm-Pollak inequality indices have the potential to inform all three questions posed in Section 2. They can provide a concise snapshot the dispersion of environmental outcomes for baseline and policy scenarios, both within and across population subgroups. In terms of ranking outcomes, they can be used to determine whether policy alternatives improve the dispersion of outcomes, holding the total amount of the outcome constant. For good outcomes the social evaluation functions associated with both indices can also be used to rank alternatives for which both the dispersion and total amount of pollution vary. Only the Kolm-Pollak index appears suitable for evaluation of bad outcomes, however.

## 5. Conclusions

For at least the past thirty years, the academic literature has used a variety of methods for quantifying the relationship between environmental quality and vulnerable sub-populations. In general, methods have been chosen with respect to their usefulness in answering questions posed by a particular study. As a result, there has been little attempt to develop a consistent framework to be used across studies, much less one suitable for the questions likely to be important for regulatory analysis. While use of a common environmental justice metric would be convenient for making comparisons and drawing conclusions across academic studies, it is essential for undertaking regulatory impact analysis in a consistent and transparent manner across different rules. In this section we discuss how well the tools presented in Sections 3 and 4 address the questions for regulatory EJ analysis posed in Section 2.

Visual displays, whether GIS maps, Lorenz curves, or concentration curves have the advantage of illuminating sharp disparities. Maps, for example, can be effective at indicating situations in which pollution levels are highly concentrated in locations with large numbers of residents belonging to vulnerable subpopulations. They are less useful for analysis of alternatives in which differences are less pronounced and not obvious to the naked eye. Nor do they suggest a means of ranking tradeoffs between total pollution reductions and reductions in disparities. Similarly, Lorenz and concentration curves are most helpful when there are sharp differences in policy options. They are not as informative if policy alternatives generate curves that cross. In general, visual displays have the disadvantage that they are not easily comparable across many alternatives, whether for an analysis of several options for implementing a given rule, or a comprehensive analysis across rules.

Subgroup summary statistics such as mean exposure rates have the advantage of being simple to calculate and easily understood. They provide useful information regarding baseline conditions, potentially providing a signal if vulnerable subgroups are more highly exposed.

These statistics have two important shortcomings, however. First, they do not provide detailed information regarding distribution of outcomes within a group. This information can be important since the impact of a pollutant may be more of a concern if it is concentrated in a hotspot among a relatively small group of individuals than if it is evenly spread across the sub-population. Second, they do not provide a clear ranking of alternatives in a systematic way. Different policy options may involve tradeoffs between total improvements across all groups and reducing the disparities among some groups. Simple averages do not provide a transparent way to resolve these conflicts.

Regression analysis can be effective in determining causality (e.g., if race is a determining factor in pollution exposure). This approach can be useful for identifying existing baseline disparities and for conducting retrospective studies. It does not appear to be well suited, however, for ranking impacts of hypothetical regulatory options.

Inequality indices seem to be a promising tool for addressing all three questions posed in Section 2. They provide a means of evaluating the distribution of environmental outcomes both within and across subgroups at baseline. Inequality indices can use model simulation results to predict distributional effects of various regulatory alternatives. Moreover, due to their associated social evaluation functions, they provide a transparent and consistent means of ranking alternatives for which both total pollution levels and their relative distributions vary. They do so at the cost of imposing restrictive value judgments on the analysis, especially with respect to the level of inequality aversion. Sensitivity analysis over a range of inequality aversion parameter values can moderate this normative influence.

Inequality indices have the advantage of a robust theoretical literature describing their properties as well as many practical applications in the context of income distribution analysis. Two of the most commonly used indices in that context, the Gini coefficient and the Atkinson index, have undesirable theoretical properties if used to measure the distributions of a “bad” like pollution, rather than a “good” like income. Specifically, the corresponding social evaluation functions are not well behaved, thus invalidating their potential for ranking options that have different tradeoffs between total improvements and reducing disparities. The concentration index, commonly used to evaluate health outcomes by income levels, has a relatively weak theoretical foundation; the corresponding social evaluation function is not as well understood. Perhaps more importantly for EJ analysis, however, is its inability to evaluate distributions across subpopulations that are not defined by income.

In contrast, the Kolm-Pollak index shares desirable theoretical traits of the Atkinson index while being able to accommodate evaluation of distributions of bads. In contrast with the other indices, however, it has a thin record of empirical applications in the context of income distribution and, to our knowledge, no published applications in the context of environmental outcomes.

Where does this leave the analyst in terms of determining a consistent and transparent method for evaluating distributional effects in regulatory analysis? Inequality indices show potential for meeting the needs of consistency in a regulatory analysis. Data are likely to be available across regulatory settings to estimate a Kolm-Pollak index, which shows the most promise for evaluating adverse environmental outcomes. This index could thus enable the decision maker to evaluate EJ consistently for a variety of rules. In addition, visual displays, summary statistics, and regression analysis provide useful supplementary information that can contribute to a richer understanding of potential EJ issues than a set of index numbers alone.

The two main impediments to using a Kolm-Pollak index in an EJ component of regulatory analysis are the lack of peer-reviewed applications and its lack of familiarity among policy-makers and the public. For it to become a useful policy tool, both of these issues need to be addressed by further academic research and pilot applications. Research regarding an appropriate range of values for the inequality aversion parameter is particularly important. This research may involve initial costs associated with both mastering practical techniques involved in its calculation, as well as costs to the user in terms of understanding the output. Such costs are likely to be small, however, compared to the relative advantage of a better understanding the distributional effects of environmental policy.
